# A case of recurrent sterile abscesses following tetanus-diphtheria vaccination treated with corticosteroids

**DOI:** 10.1186/s12879-020-05756-3

**Published:** 2021-01-12

**Authors:** Abdurrahman Kaya, Sibel Yıldız Kaya

**Affiliations:** 1 Department of Infectious Diseases, İstanbul Training and Research Hospital, Istanbul, Turkey; 2 Infectious Diseases Unit, Sungurlu State Hospital, Çorum, Turkey

**Keywords:** Sterile abscess, Vaccination, Tetanus

## Abstract

**Background:**

Vaccinations have been widely used worldwide since their invention to prevent various diseases, but they can also have some adverse effects ranging from mild local reactions to serious side effects. These adverse effects are generally self-limited and resolve within a short time without any treatment. While a sterile abscess following vaccination is a rare condition in adults, many cases have been reported regarding children in the literature. Here, we report a case of recurrent sterile abscesses, which occurred after a Td vaccination, treated with corticosteroids.

**Case presentation:**

A 22-year old woman was admitted to our department with a complaint of swelling at the site of the vaccination. On physical examination, this mass was about 6 × 6 cm in size and fluctuating, but there were no pain complaints and no redness present. She had received her Td vaccination 3 weeks ago and the swelling had started at the site of the injection 4 days following this immunization. Oral amoxicillin/clavulanic acid and local antibiotic cream were administered for 10 days. The laboratory values were unremarkable. Despite the administration of antibiotics, the swelling did not regress, and on the contrary, continued to increase in size. On ultrasound, two interconnected abscesses were observed in the subcutaneous area, and did not involve the muscle tissue. Later, the abscesses were completely drained, and the samples were cultured. The current antibiotics were continued. The gram staining of the samples revealed abundant leukocytes but no microorganisms. The solid and liquid cultures of the materials remained negative. Despite the administration of multiple drainages and antibiotics, the mass recurred. Finally, the patient was considered to have a sterile abscess due to Td immunization. The antimicrobials were stopped. Local and oral corticosteroids were initiated. The swelling regressed significantly, and the treatments continued for 7 days. The patient has been doing well and has had no recurrence for over a year.

**Conclusions:**

Corticosteroids appeared to improve the patient and therefore we suggest that the efficacy and route of administration of steroids in this situation should be explored further.

## Background

Tetanus is a severe life-threatening disease caused by Clostridium Tetani and can be prevented through immunization with tetanus-toxoid-containing vaccines. These vaccinations have been widely administered in both children and adults. They have some adverse effects ranging from mild local reactions such as pain, tenderness, and swelling, to serious side effects such as seizures and acute encephalopathy [1, 2]. While a sterile abscess following vaccination is a rare condition in adults, many cases have been reported in children in the literature [3–7]. Here, we report a case of recurrent sterile abscess following Tetanus-diphtheria (Td) vaccination in an immunocompetent host.

## Case presentation

**Fig. 1:**
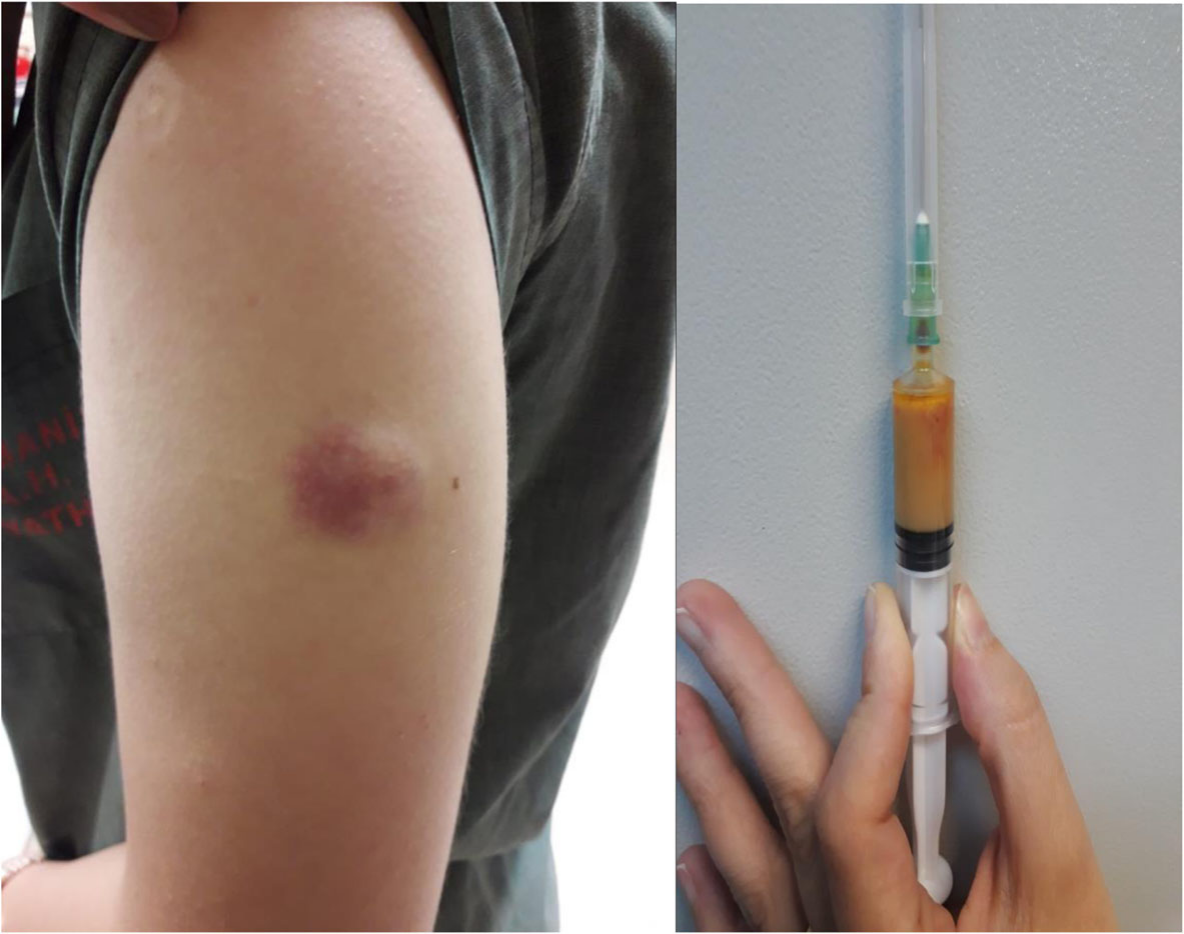
Fluctuant and painless mass without warmth and erythema

**Fig. 2:**
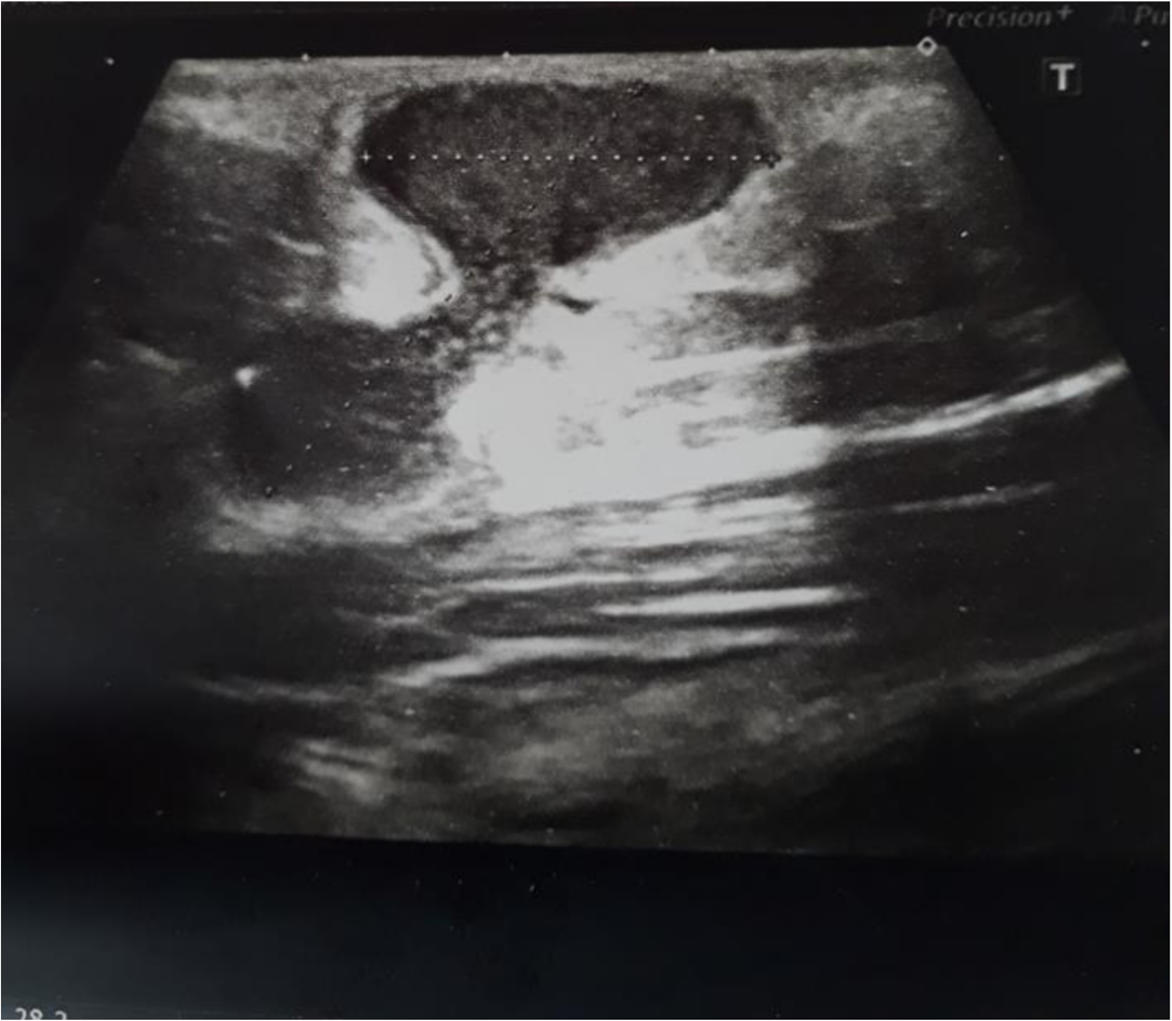
Two interconnected abscesses in the subcutaneous area

A 22-year-old female patient was admitted to our clinic with swelling at the site of the vaccination. She had received her Td vaccination for internship 3 weeks ago, and the swelling had started at the injection site 4 days following this immunization. The vaccine was a toxoid vaccine containing aluminum adjuvant (Tedatif; Turk Drug and Serum Industry Inc). On physical examination, this mass was about 6 × 6 cm in size and fluctuating, but had no pain, and no redness (Fig. 1**)**. Oral amoxicillin/clavulanic acid (daily 2 × 1 gram) and local antibiotic (2% cream, Fusidic acid 2 × 1) were applied for 10 days. She had no immunosuppressive disease and no history of recurrent infection. On her medical history, she had received all of her childhood vaccinations and had not experienced any serious side effects. During admission, the patient had no history of trauma, and no fever, and no lymphadenopathy on examination. The laboratory values showed a total white blood cells count of 6500 cells/ McL, with 70% neutrophils, while C3, C4, immunoglobulin (Ig) A, IgM, IgG, IgE, sedimentation rate, and C-reactive protein were within normal ranges. Serology for human immunodeficiency virus was negative. On ultrasound, two interconnected abscesses were seen in the subcutaneous area, and did not involve the muscle tissue **(**Fig. 2**)**. She was not pregnant and did not receive any other vaccines simultaneously. Despite the administration of antibiotics, the swelling did not regress, and on the contrary, continued to increase in size. Later, the abscesses were completely drained, and the samples were cultured. The current antibiotics were continued. The gram staining of the samples revealed abundant leukocytes but no microorganisms. The solid and liquid cultures of the materials remained negative, although they were repeated more than once. Under the current antibiotic treatments, the swelling appeared again at the same site 6 days after the first draining. Then the mass was re-drained. The cultures of samples remained negative and no specific etiology was reached. However, seven days after the second draining, the mass re-appeared. Following the drainage, the abscesses were cultured. The abscess aspirate was not cultured for tuberculous bacilli. The Ehrlich-Ziehl-Neelsen of the sample was negative and other results including cultures and gram staining of the specimens remained negative. After a course of 23 days of treatment, the causal etiology was not identified. Finally, the patient was regarded as having a sterile abscess due to the Td immunization. The antimicrobials were stopped and oral prednisolone (daily 40 mg) and mometasone furoate (0.1% cream, 2 × 1) were initiated. The swelling regressed significantly, and the treatments continued for seven days. The patient has been doing well and no recurrence has been observed for over a year.

## Discussion and conclusions

With common, modern, and safe vaccines, although the incidence of vaccine-preventable diseases has decreased significantly, vaccination can still give rise to some undesirable side effects. These adverse effects generally are self-limited and they get resolved in a short period of time without any treatment [8]. However, in this case, the mass recurred for more than 3 weeks and its recurrence was atypical and extraordinary. Moreover, although previous cases of a single abscess had been reported, interestingly our case had two interconnected abscesses. Since the majority of vaccinations are administered in children, adverse events are commonly seen in these populations. These events have been more frequently reported in females than males following diphtheria, tetanus, and pertussis (DTaP) vaccinations [9–11]. In our case, the patient was an adult female, and she denied any past adverse reaction following any previous immunization. The case report patient also received a different type of vaccine Td not including pertussis.

A sterile abscess is a condition in which infectious etiology cannot be shown or it is defined as a type IV hypersensitivity reaction to vaccination in literature, but there is only limited data available supporting delayed-type hypersensitivity [12]. Sterile abscess formation following DTaP immunization very rarely occurs in 6–10 per million doses of DTaP vaccination [13]. Generally, fever and lymphadenopathy do not accompany a sterile abscess, and the pus remains negative for infectious etiology with gram staining, culture, and other tests [12]. Most abscesses at the site of vaccination are caused by infection and generally, a combination of drainage and antibiotics is initially administered for treatment [14]. Yet, her mass failed to improve on the combination of these treatments. On the other hand, gram staining is a test that gives an early indication of potential bacteria through visualization of the bacteria and not affected by prior antibiotic use. Therefore, as recommended by Wise et.al., it was included in the diagnostic test for making the distinction between an infectious abscess and a sterile abscess in 2007 [12]. Also, in such recurrent abscesses; generally infectious etiology was not found, and the patients were finally diagnosed with sterile abscesses in many studies [3–7, 11, 15]. Therefore, a sterile abscess should be considered in such cases.

**Fig. 3:**
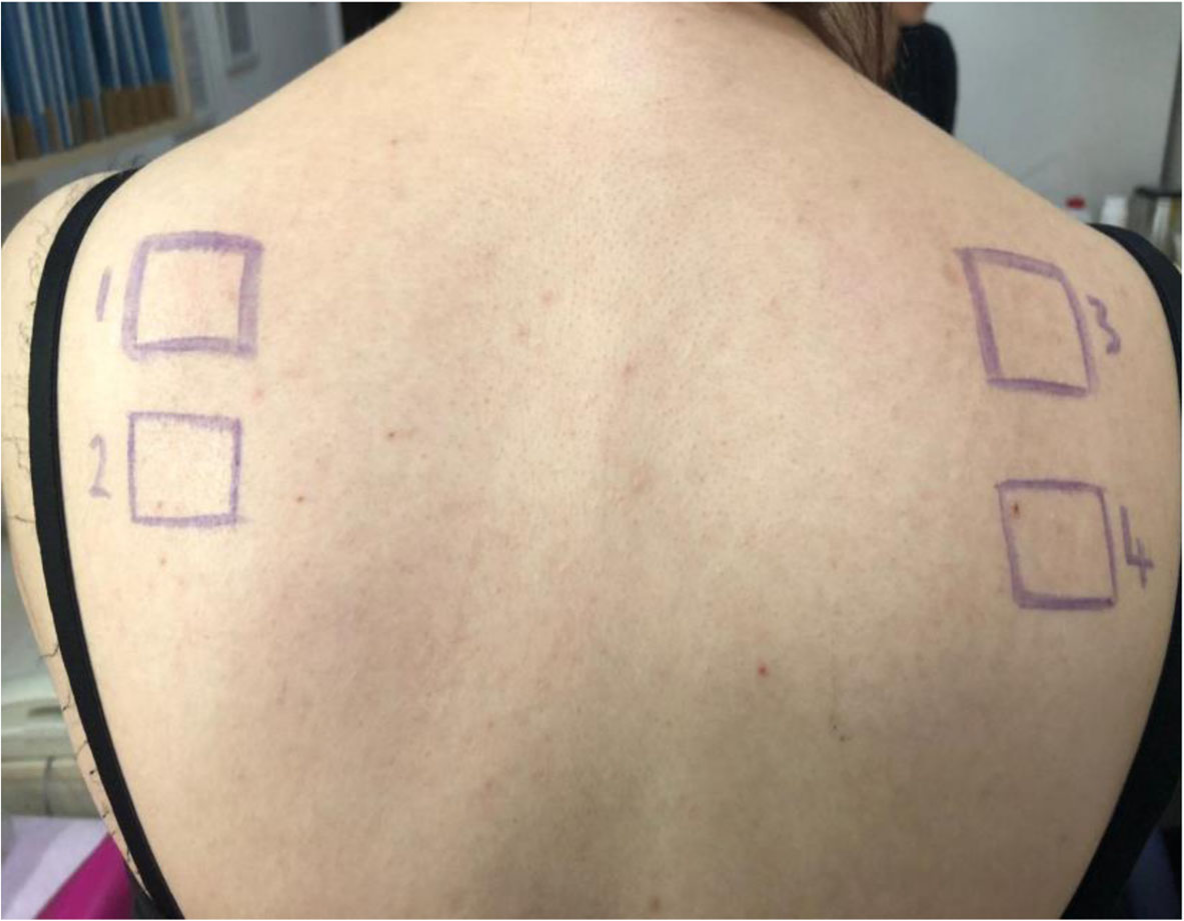
Patch testing to the vaccine components

The pathogenesis of sterile abscess following vaccination is not well elucidated and little information is found on the etiology, the optimal evaluation, and the treatment of this entity [12]. Some studies have reported about injection site abscesses after vaccinations and stated many different causes including bacterial contamination of the vials or syringes, faulty technique, and hypersensitivity reaction to high level of aluminum adjuvant [8, 16, 17]. Most inactivated vaccines contain an adjuvant and are administered intramuscularly. An adjuvant is a vaccine component and can cause an exaggerated local reaction if not injected into the muscle [11, 18]. Proper injection technique is a critical component of a successful immunization. Needles must be sterile, and disposable, and also a separate needle and syringe should be used for each injection. Inappropriate needle selection, aspiration prior to injection of the vaccine, and wrong injection route are among the faulty administration techniques [19]. Adverse events are more common if a vaccine is injected subcutaneously instead of intramuscularly. Our patient received an inactivated vaccine containing aluminum adjuvant. Her abscesses were located in the subcutaneous area and the muscle tissue was not involved. Therefore, a sterile abscess could have resulted from misinjection in our case. Also, it has been suggested that recurrent sterile abscesses are primarily associated with aluminum-adsorbed vaccines [14, 18]. Aluminum is commonly used as an adjuvant in many vaccines, and facilitates a delayed release of antigen at the site of injection, thus prolonging contact with antigen-presenting cells [20]. This may lead to an accumulation of macrophages and immune cells at the site of injection, which may incidentally trigger the formation of sterile abscesses [20]. In the present case, aluminum, formaldehyde, 2-phenoxyethanol, and thimerosal were considered as possible causes of a sterile abscess. Patch testing was performed but remained negative for all of them (Fig. 3). In the literature, sterile abscesses were treated with drainage [7, 11, 15, 21, 22]. Our patient is the first case reported who was treated with corticosteroids. As corticosteroids are used in the treatment of inflammatory, allergic, and immunologic disorders; they were administered to the patient in order to reduce inflammation, but subsequently caused a complete cure.

As a result, corticosteroids appeared to improve the patient and therefore we suggest that the efficacy and route of administration of steroids in this situation should be explored further.

## Data Availability

Not applicable.

## Abbreviations

DTaP: Diphtheria, tetanus and pertussis
Ig: Immunoglobulin
Td: Tetanus-diphtheria
